# Mapping whole brain effects of infrared neural stimulation with positron emission tomography

**DOI:** 10.1162/imag_a_00052

**Published:** 2023-12-21

**Authors:** Marcello Meneghetti, Frederik Gudmundsen, Naja S. Jessen, Kunyang Sui, Christina Baun, Mikael Palner, Christos Markos

**Affiliations:** Department of Electrical and Photonics Engineering, Technical University of Denmark, Lyngby, Denmark; Department of Neuroscience, University of Copenhagen, Copenhagen, Denmark; Department of Clinical Research, Clinical Physiology and Nuclear Medicine, University of Southern Denmark,Odense, Denmark; Department of Nuclear Medicine, Odense University Hospital, Copenhagen, Denmark; Department of Drug Design and Pharmacology, University of Copenhagen, Odense, Denmark; Neurobiology Research Unit, Copenhagen University Hospital, Copenhagen, Denmark; NORBLIS ApS, Virum, Denmark

**Keywords:** INS, PET, corticostriatal circuits, polymer optical fibers

## Abstract

The combination of neuroimaging and targeted neuromodulation is a crucial tool to gain a deeper understanding of neural networks at a circuit level. Infrared neurostimulation (INS) is a promising optical modality that allows to evoke neuronal activity with high spatial resolution without need for the introduction of exogenous substances in the brain. Here, we report the use of whole-brain functional [^18^F]fluorodeoxyglucose positron emission tomography (FDG-PET) imaging during INS in the dorsal striatum, performed using a multifunctional soft neural probe. We demonstrate the possibility to identify multi-circuit connection patterns in both cortical and subcortical brain regions within a single scan. By using a bolus plus infusion FDG-PET scanning protocol, we were able to observe the metabolic rate evolution in these regions during the experiments and correlate its variation with the onset of the INS stimulus. Due to the focality of INS and the large amount of viable molecular targets for positron emission tomography (PET), this novel approach to simultaneous imaging and stimulation is highly versatile. This pilot study can pave the way to further understand the brain connectivity on a global scale.

## 1 Introduction

Understanding the neural functional networks is a crucial goal for the advance of neuroscience and for the development of new treatments for brain diseases (Vázquez-Guardado et al., 2020). A novel tool towards this goal is the combination of neuromodulation techniques with whole-brain functional neuroimaging to characterize regional activity both locally and distally (Tolias et al., 2005). Several studies have been already produced combining invasive modulation techniques such as deep brain stimulation (DBS), optogenetic stimulation and chemogenetics, as well as non-invasive ones like focused ultrasound stimulation (FUS) and transcranial direct current stimulation (TDCS), with functional magnetic resonance imaging (fMRI) and positron emission tomography (PET), showing that this approach is highly promising (Casado-Sainz et al., 2022;He et al., 2019;Kim et al., 2013;Klein et al., 2011;Le Jeune et al., 2010;Lee et al., 2010;Min et al., 2012;Rudroff et al., 2020;Thanos et al., 2013;Weitz et al., 2015;Zhao et al., 2020). However, within the invasive techniques, lack of cell-type and spatial specificity, together with the development of scar tissue around chronic implants and complications arising from parasitic Joule heating are well-known issues with electrical stimulation (Won et al., 2020). Optogenetics and chemogenetics excel in cell-type specificity, but they suffer from intrinsic added complexity given by the requirement of genetic manipulation of neurons by either viral injection or use of genetically modified animals. Therefore, their translation to clinical use will require further refinement of the genetic toolbox and of the delivery of transgenic tools (Bansal et al., 2022;Boehm et al., 2021). Furthermore, the short light wavelengths typically used in optogenetics can generate Becquerel-effect-induced photoelectric artifacts when combined with electrophysiology (Bernstein et al., 2012;Jiang et al., 2022;Kozai & Vazquez, 2015). Due to these reasons, infrared neural stimulation (INS) has recently gained traction as an alternative focal stimulation technique which can be used both as a non-invasive technique at superficial regions, or through small optic fiber implant with high spatial resolution (Salehpour et al., 2018). This further enables deep structural targeting and simultaneous electrophysiological measurements (Tan et al., 2015). The underlying mechanism of INS relies on thermally mediated biological processes that are directly induced by the absorption of infrared (IR) light by biological tissues, and as such no introduction of exogenous substances in the brain is required (J. Wells et al., 2007). The exact biological mechanism allowing the IR-induced temperature raise in neurons to evoke activity is still a matter of debate. Two of the most often proposed hypotheses are changes in the in-membrane capacitance induced by fast heat gradients (Liljemalm et al., 2013;Shapiro et al., 2012) and involvement of temperature-sensitive receptors (Albert et al., 2012;Chernov & Roe, 2014;Suh et al., 2009;Yao et al., 2009). Moreover, by varying the operational wavelength within the water absorption spectrum, the penetration length of the stimulation light can be tuned, allowing for spatial resolution down to the individual axon scale (Cayce et al., 2015;J. D. Wells et al., 2005). This focal nature of the INS stimulus can, when the correct optical parameter are chosen, be used to target submillimeter domains and small neural sub-circuits despite its lack of cell specificity (Xu et al., 2019). In recent years, the combination of INS and fMRI has been proven to be a powerful tool to study the whole-brain functional connectome, with distinct advantages in terms of speed and resolution (Shi et al., 2021;Xu et al., 2019).

Between neuroimaging techniques, PET has an unique strength in terms of breadth of applications, as the increasing diversity of radiotracers allows for a wide number of imageable targets and types of measurable outcomes (Hooker & Carson, 2019). The use of [^18^F]fluorodeoxyglucose as a radiotracer (FDG-PET), in particular, is the most direct technique to image cerebral metabolism of glucose in the brain, which directly reflects neuronal and glial activity (Lundgaard et al., 2015;Verger & Guedj, 2018). The introduction of microPET scanners with high sensitivity and accuracy further increased the potential of FDG-PET as an investigation tool for pre-clinical studies (Kuang et al., 2020). Furthermore, FDG-PET has the potential to effectively provide information on the brain metabolism in awake small animals. If FDG (i.p.) was to be injected prior to neuromodulation in an awake and behaving animal, and the PET scan conducted following the end of the FDG uptake period, then a snapshot of the total uptake changes doing the modulation and behavior sessions could be measured, similarly to what we and others have previously done using chemogenetics or optogenetics as a modulation technique (Casado-Sainz et al., 2022;Thanos et al., 2013). For applications in combination with INS, FDG-PET also has a distinct advantage over blood oxygenation level dependent (BOLD) fMRI, as resonance frequency shifts due to INS-induced temperature rising might generate non-neural components in the BOLD signal at the stimulation site (Casado-Sainz et al., 2022;Shi et al., 2021). Moreover, FDG-PET is not hindered by the presence of metallic implants (De Winter et al., 2003). This is crucial in enabling precise experiments combining whole-brain imaging with local measurements of neuronal activity through electrophysiology during INS.

Electrophysiology is a well-known and established technique for studying neural activity with high sensitivity, temporal resolution, and signal-to-noise ratio (SNR), and shares with INS the strength of not needing viral or chemical injections of “translators” (Scanziani & Häusser, 2009). The combination of INS and electrophysiology*in vivo*in both acute and chronic experimental settings is further facilitated by the recent development of multifunctional implantable neural interfaces that are able to simultaneously deliver IR light in subcortical brain regions and record electrical signals (Horváth et al., 2020;Meneghetti et al., 2023). Soft implantable devices, in particular, can enable the interrogation of brain circuitry in chronic settings with reduced inflammation and glial scarring while simplifying surgical procedures required for simultaneous multimodal studies by integrating different functionalities such as stimulation, recording, drug delivery, and temperature sensing (Meneghetti et al., 2023;Park et al., 2019;Sui, Ioannou, et al., 2023;Sui, Meneghetti, Berg, et al., 2023;Sui, Meneghetti, Li, et al., 2023;Won et al., 2020).

Because of all these factors, the combination of INS and PET is a powerful tool in neuroscience, allowing a broad range of complex*in vivo*experiments for the study of brain function in behaving animals on a scale ranging from the single axon to the whole brain.

**Fig. 1.**
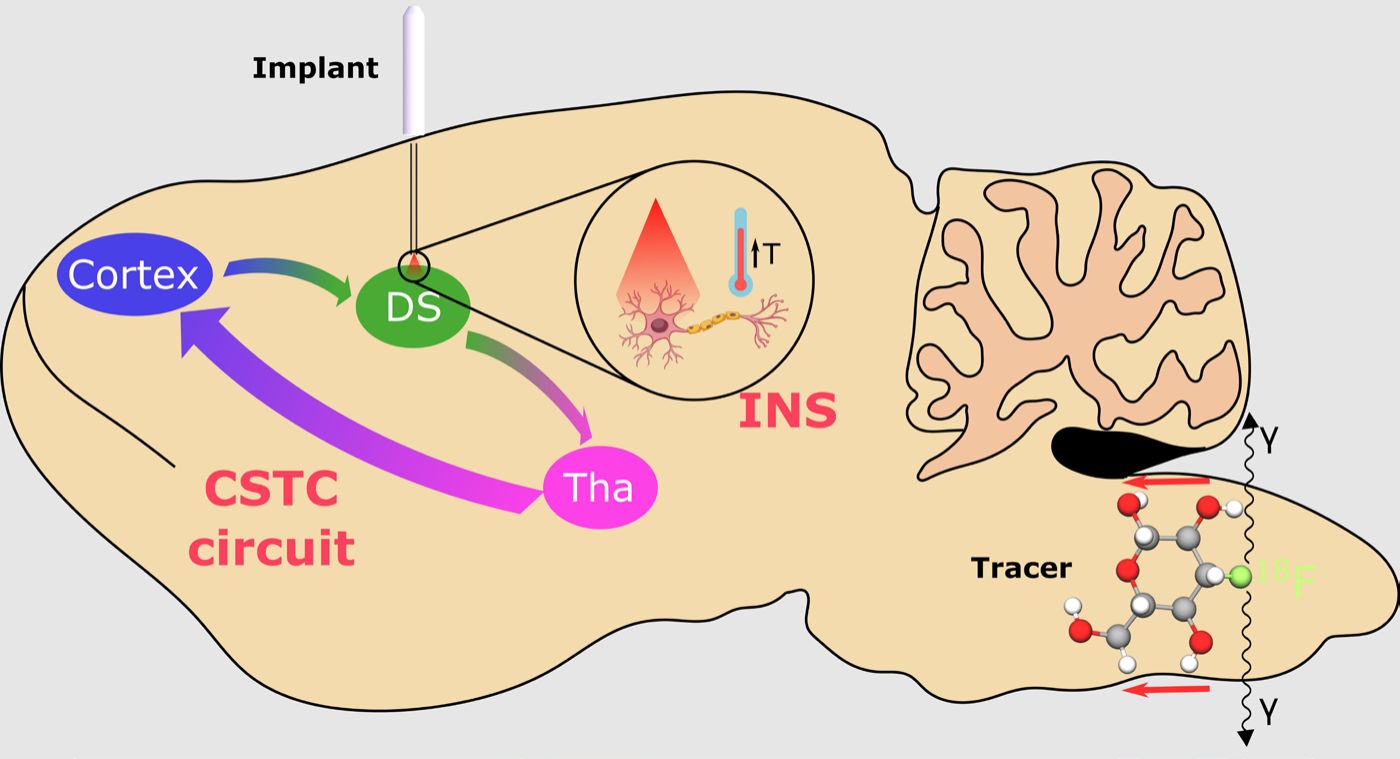
Schematic representation of the study: infrared neurostimulation has been applied to the dorsal striatum using a multifuntional neural interface during the infusion of FDG, and its effects on the CSTC circuits have been evaluated by PET. Tha: thalamus, DS: dorsal striatum

In this proof-of-principle study, we combine whole-brain dynamic FDG-PET with INS stimulation in the dorsal striatum (DS), similar to our previous target using chemogenetics (Casado-Sainz et al., 2022). We chose this target as a study case to demonstrate for the first time the combined use of INS and PET for mapping neural circuits*in vivo*(Fig. 1). This allowed us to benchmark the effect of the stimulation on the connectivity within the cortico-striatal-thalamic-cortical (CSTC) circuit, which has been widely studied due to its major involvement in motor disorders such as Parkinson’s disease and Tourette syndrome (Raval et al., 2021;Sowell et al., 2008;Vriend et al., 2014) and behavioral disorders such as obsessive-compulsive disorder and attention deficit hyperactivity disorder (Ting & Feng, 2011;Zhu et al., 2018). To provide temporal correlation between the stimulation and the metabolism variation, the tracer was slowly infused during the scans to add a temporal dimension to the PET results. The INS stimulus has been delivered using an advanced monolithic fiber interface capable of simultaneous optical stimulation and electrical recording. This soft and biocompatible device, developed by our group, allows to deliver light in the high-water-absorption spectral region inside the brain tissue while recording an electrophysiological signal with single-unit resolution (Meneghetti et al., 2023).

## 2 Materials and Methods

### 2.1 Neural implants

**Fig. 2.**
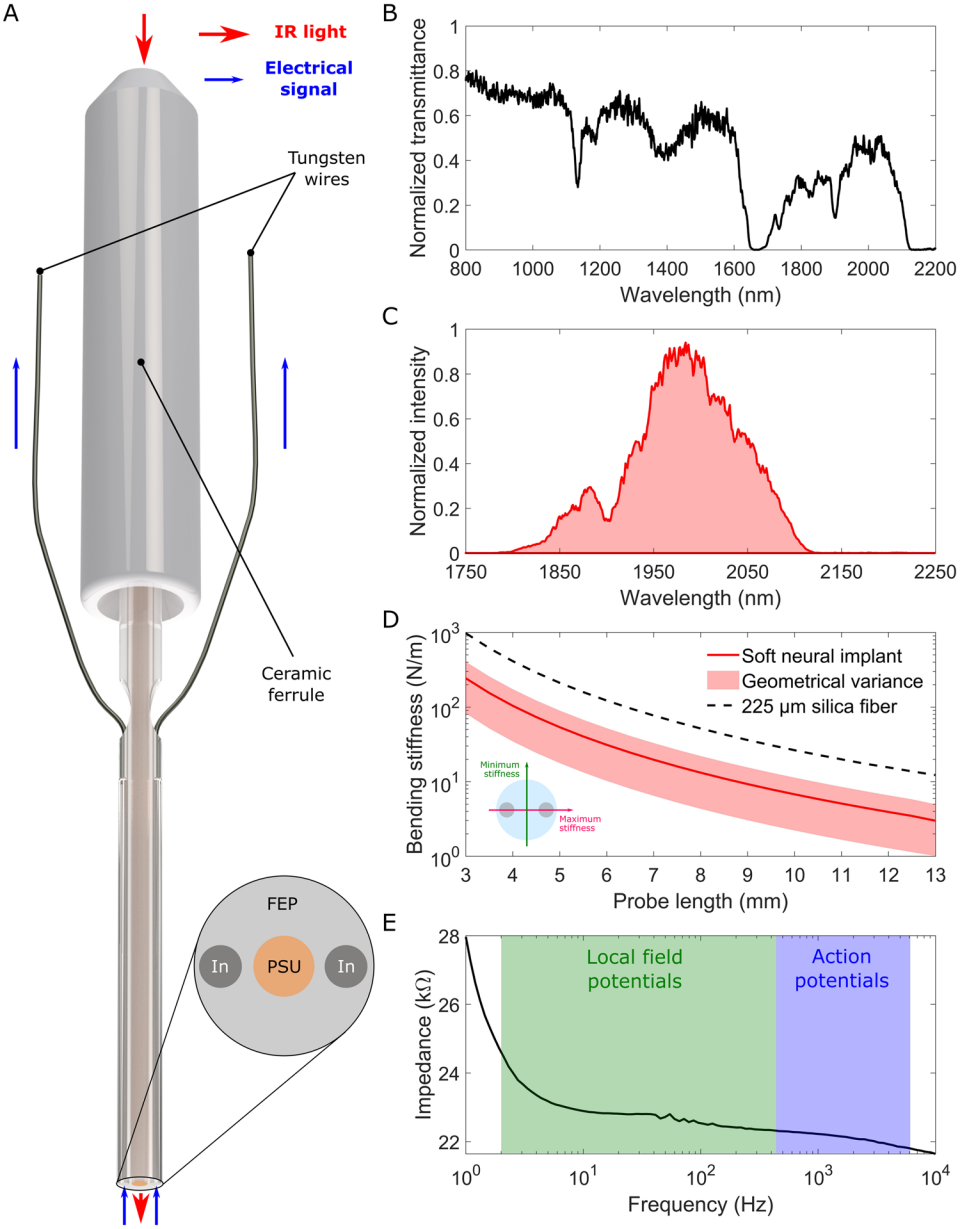
(A) Schematic representation of an electrophysiology-eneabled neural interface based on a polymer optical fiber (POF) with a polysulfone (PSU) core and fluorinated ethylene propylene (FEP) cladding, functionalized with indium electrodes. (B) Normalized infrared transmittance spectrum of the POF. (C) Normalized output light spectrum of a neural interface connected to the INM setup. (D) Comparison between the average bending stiffness (solid line) of our custom soft neural implant and the one of a standard 225μmsilica fiber; the shaded area around the soft probe stiffness represents the minimum and maximum values corresponding to the two main axes of the fiber. (E) Typical impedance spectrum of one of the indium electrodes integrated in our probes.

**Fig. 3.**
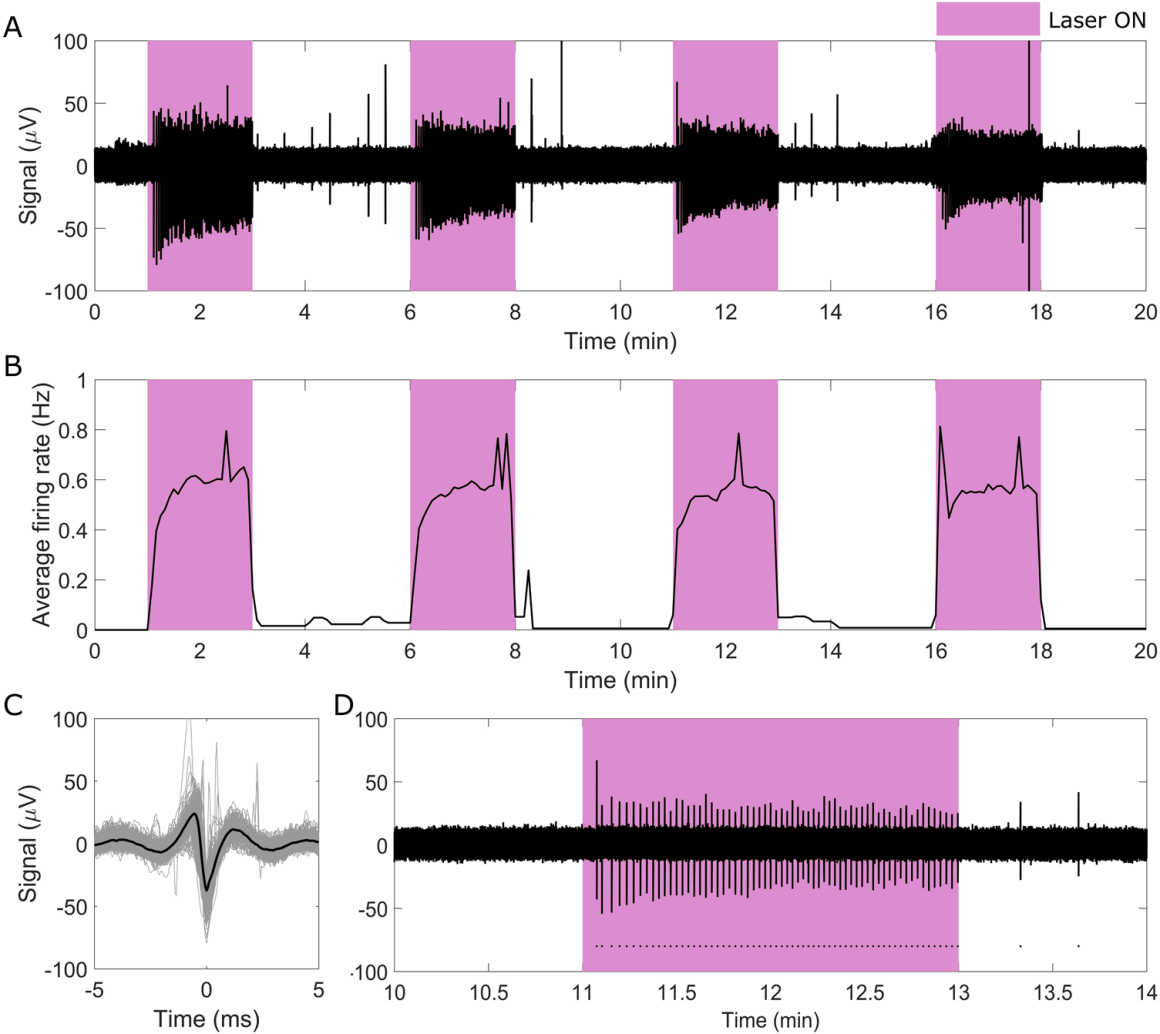
(A) Electrophysiological recording performed over several cycles of INS (2 minute ON/3 minute OFF stimulation). (B) Average firing rate measured during the stimulation (5 second bins). (C) Overlap (grey) and average (black) of all the recorded spikes. (D) Zoom of a single stimulation period, with dots highliting the temporal location of individual spikes.

In this study, we used soft infrared neural implants developed by our group for chronic INS applications (Meneghetti et al., 2023) (Fig. 2A). These implants, based on a polymer optical fiber (POF) with a core diameter of~105µm, were fabricated by the thermal drawing process. Polysulfone (PSU) was used as a core material to achieve enhanced IR light transmission up to 2100 nm wavelength (Fig. 2B, C), while fluorinated ethylene propylene (FEP) was used as a cladding. The use of FEP as a cladding strongly reduced the bending stiffness of the probes with respect to standard silica glass fibers, enhancing their long-term biocompatibility (Fig. 2D) (Sui et al., 2022). Hybrid indium-tungsten electrodes enable electrophysiological recording at the fiber tip. The electrodes have an impedance of<35 kΩin the 1-10,000 Hz range (the impedance spectrum of one of the electrodes is shown inFig. 2E), and have been proven to be suitable for*in vivo*extracellular electrophysiology during INS experiments performed with 2 minute stimulations interspersed by 3 minutes resting periods (Fig. 3). The implants are connectorized with a standard optical fiber ferrule (1.25 mm LC/PC). The implantable length of the interfaces was set at 7 mm for the DS.

### 2.2 Animals

Six male wild-type adult Long Evans rats (300±50g for rats in the INS group,335±13g for rats in the baseline group) were used for the*in vivo*experimentation. Three of the animals underwent stereotaxic surgery for probe implantation before the PET/CT scans and were used for the INS experiments (INS group), while the other three underwent the PET/CT scans using the same protocol but without prior surgery or stimulation to act as a control group (baseline group). All the procedures performed have been approved by the Animal Experiments Inspectorate under the Danish Ministry of Food, Agriculture, and Fisheries, and in compliance with the European guidelines for the care and use of laboratory animals, EU directive 2010/63/EU.

### 2.3 Stereotaxic surgery

The animals were all fasted overnight before the scans to ensure low blood sugar and maximal uptake of [^18^F]FDG. During all the procedures, anesthesia was maintained using 2% isoflurane in 100% oxygen. Prior to the incision, lidocaine was administered subcutaneously at the site, and ocryl gel was applied to keep the eye moisture during the procedure. Then, the head of the animal was mounted into the stereotaxic frame, where a heating pad was placed below the animal to maintain the body temperature throughout the surgery. A median incision was performed in the scalp to expose the skull, which was then cleared from connective tissue and thoroughly washed by successive applications of hydrogen peroxide and saline solution. A 1 mm diameter hole was drilled at the target location using an electric dental drill. After the drilling, the dura layer was removed. To avoid any damage to the brain, this operation was carefully performed with fine forceps. Once the brain tissue was exposed, the neural implant was fixed to the arm of the stereotaxic frame and slowly inserted into the brain. Finally, the implant was fixed in place with dental cement to prevent any probe movement during the scanning. The stereotaxic coordinates used for the implantations were the following: anterioposterior 1.1 mm, mediolateral 2 mm, and dorsoventral 4 mm.

### 2.4 Tracer preparation and administration

The [^18^F]FDG used in this work was from the daily preparation for clinical use (Department of Nuclear Medicine, Odense University Hospital).

### 2.5 PET/CT scan protocol

Following the acute implantation of the neural implant, a catheter was placed in the tail vein of the rat. The animal was then moved to the Siemens Inveon PET/SPECT/CT scanner operating in docked mode (Siemens, Knoxville, Tennessee, USA). They were placed on a heated bed and kept under light isoflurane anesthesia (1.5-2% isoflurane in 100% oxygen). Anatomical CT scans of the head were performed with the following settings: full rotation in 270-degree projections, 4 x 4 bin, and low magnification; scan length at 90 mm with an effective pixel size at 111.25 µm and exposure settings 80 kV and 500 µA in 350 ms. The CT scan was reconstructed using the Feldkamp algorithm with a Sheep-Logan filter, slight noise reduction, and Hounsfield calibration. Following the CT scan, infusion of the radiotracer was started through the vein catether by slowly injecting 140-145 MBq [^18^F]FDG in 0.64 mL using an infusion pump. A dynamic PET acquisition was started concurrently with the start of [^18^F]FDG infusion (8 uL/min). The PET scan was performed in the energy window 350-650 keV and axial scan length of 127 mm, for a total of 80 minutes uninterrupted acquisition time (40 minutes baseline, 20 minutes stimulation, 20 minutes poststimulation). The data were histogrammed into one 20 minute time frame and sixty 1 minute time frames, starting from 20 minutes, giving a total of 60 x 1 minute dynamic frames. CT and PET images were co-registered using a transformation. Reconstruction of PET data was performed using an OSEM3D/SP-MAP algorithm (2 x OSEM iterations and 18 x MAP iterations) with scatter correction and a matrix size 128 x 128, resulting in a final target resolution of 1.5 mm.

### 2.6 INS protocol

For the INS stimulus, light from a supercontinuum source (NKT SuperK, average power up to 6 W) was coupled to a 105 µm core silica patch cable after long-pass filtering with a cut-off wavelength of 1800 nm. Each of the probes was then individually connected to the patch cable using a ceramic sleeve and its output power at different laser currents was calibrated using a Thorlabs S401C thermal power sensor. Following implantation in the brain, the neural interfaces were reconnected to the patch cable after having placed the animals in the scanner. During the stimulation, light was delivered continuously for 2 minutes intervals, followed by 2 minutes resting periods.

### 2.7 Image and data analysis

Decay-corrected FDG-PET images were analyzed in PMOD version 4.2 (PMOD Technologies LLC, Zürich, Switzerland). They were cropped to contain only the head of the animal and matched using an automated pipeline to the Px Rat (W. Schiffer)-FDG-PET template (PMOD v4.2) (Schiffer et al., 2006). Here, individual time activity curves from each region of the atlas were extracted and calculated as metabolic uptake in the region divided by whole-brain uptake. Each individual time activity curve was then normalized to the baseline metabolic uptake rate during the first 20 minutes, and changes in uptake in relation to baseline plotted as a function of time. The 1 minute histogrammed PET images were time normalized in PMOD to show differences in metabolic rate over time. From one animal, two average PET images were made from 1-20 minutes and from 41-60 minutes dynamic frames; these images were then subtracted from one another to show the voxel-specific changes between baseline and poststimulation condition.

## 3 Results

### 3.1 Targeting during implantation

**Fig. 4.**
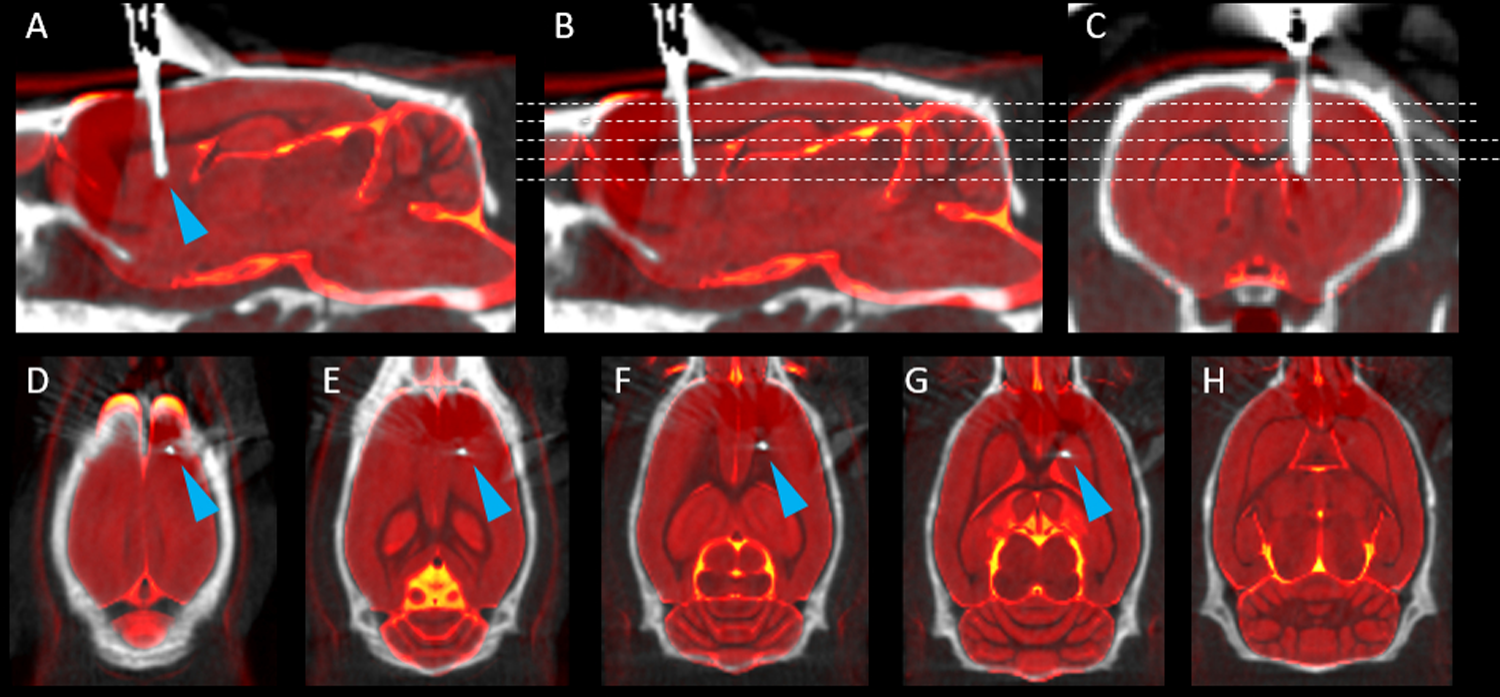
(A-C) Sagittal and coronal CT slices (greyscale) overlayed with a standard high resolution structural MR (redscale) showing the location of the neural interface (blue arrow) in the right DS. (D-H) Horizontal slices from the planes shown as dashed lines in (B and C), showing the trace of the neural interface through the brain.

When using flexible neural interfaces as probes, correctly targeting the selected brain region during implantation without the use of a guide fixture can be a challenge (Park et al., 2019). The importance of the positioning of the probe is even more relevant when using a highly localized stimulation such as the one provided by INS in the 2 µm spectral region. Here, a slow and continuous insertion was used to limit the risk of bending as the fiber penetrates the tissue. The correspondence between the fiber tip location after implantation and the desired stereotaxic coordinates was verified by localizing the fiber in an overlap of PET and CT scans prior to the beginning of the stimulation (Fig. 4). As shown inFigure 4A-C, no significant bending of the fiber with respect to the ceramic ferrule is apparent after insertion. The horizontal slices shown inFigure 4D-Hshow the path of the probe through the brain and confirm its location in the targeted region of the right DS.

### 3.2 Input function and whole-brain mapping

**Fig. 5.**
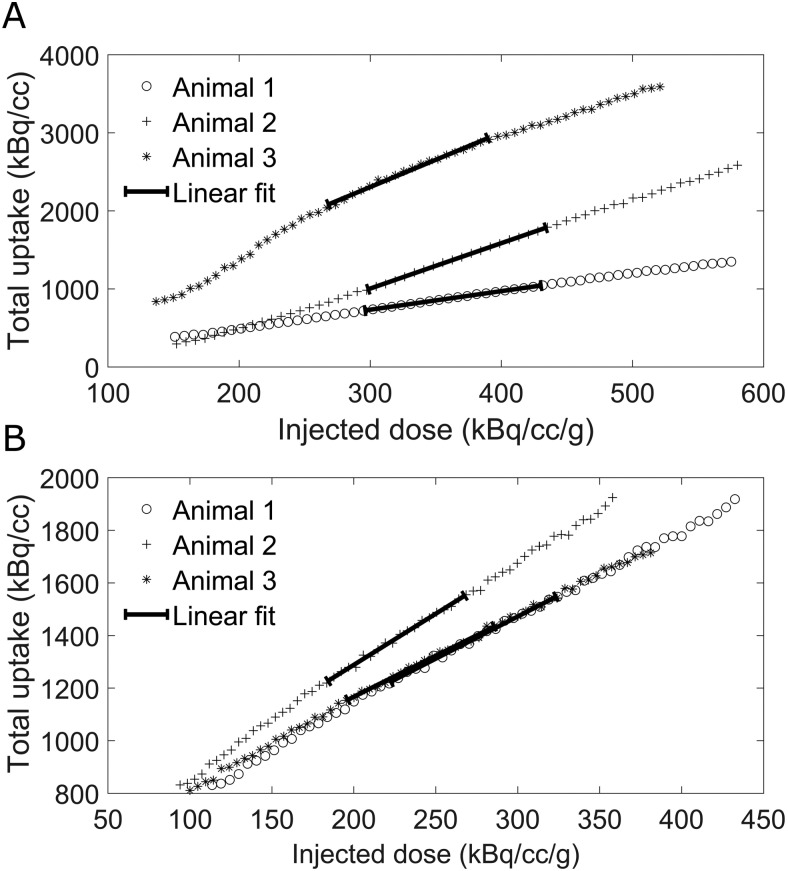
Whole-brain uptake as a function of the radiotracer infusion for (A) the three animals in the INS group and (B) the three animals in the baseline group, with linear fits of the data highlighting the period corresponding to the INS stimulus.

In order to be able to compare the activity variation between different animals, all the metabolic rate results presented in the following sections were normalized to the whole-brain uptake of radiotracer of the corresponding animal.Figure 5shows the used image derived input functions, that is, the total activity (kBq/cc) in the whole brain as a function of radiotracer infusion, for both the INS (A) and baseline (B) groups. While some of the animals slightly deviate from a purely linear behavior of the input function of infusion, this effect is limited to the first minutes of the scans, and as such does not affect the signal recorded for the periods corresponding to the stimulation. This is highlighted by the linear fits of the input function during the 20 to 40 minutes period of the scan (corresponding to the stimulation in the INS group) shown inFigure 5.

The total signal collected from the brain in one of the animals in the INS group is presented inFigure 6A-D, with A presenting the exact location of the neural interface during the scan, and B-D showing all the scans (one per minute) collected before, during, and after stimulation, respectively. The magnitude in all scans has been normalized voxel-by-voxel by the total brain uptake of radiotracer.

**Fig. 6.**
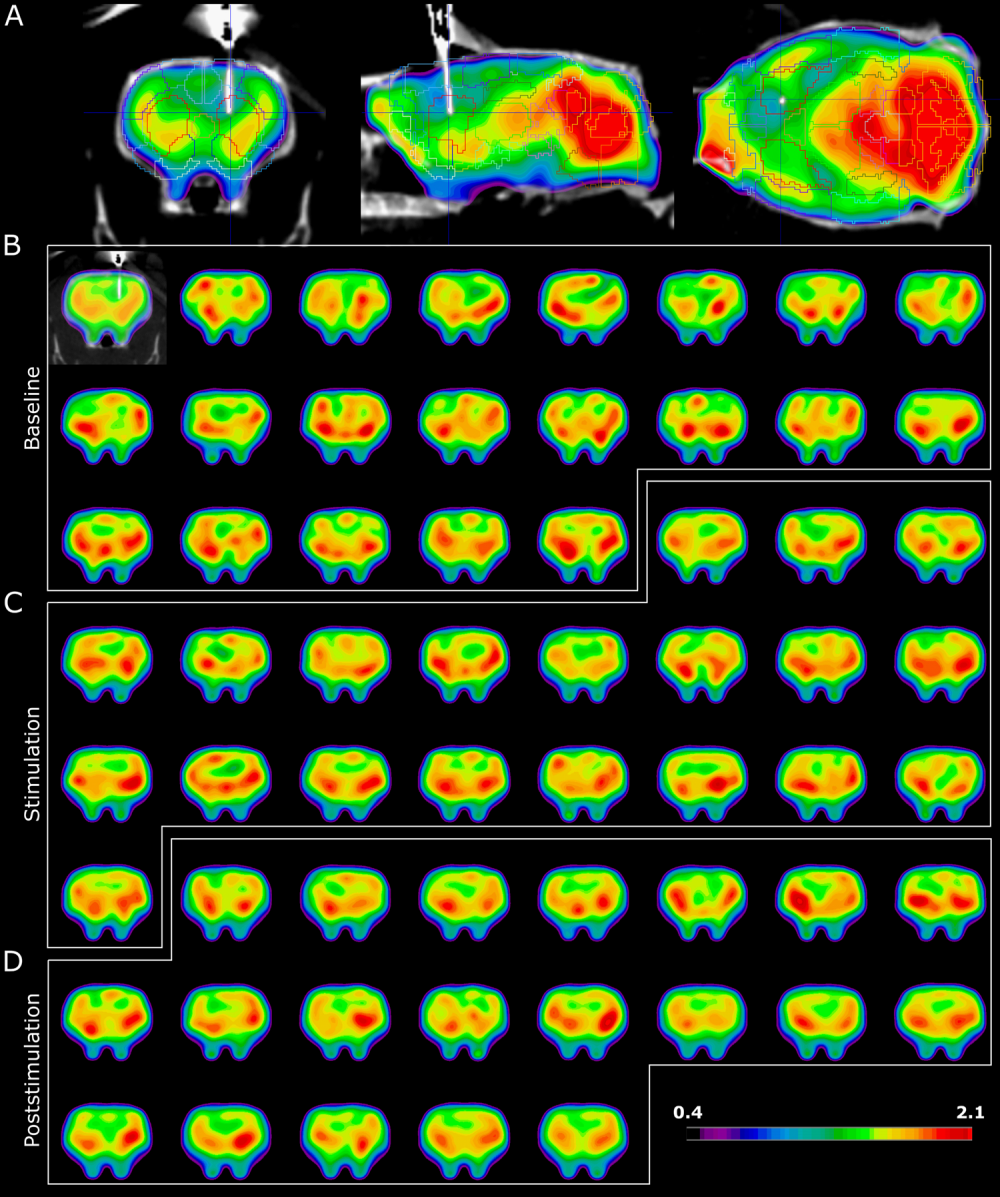
(A) Position of the fiber tip in the right DS during the scan, here shown as the first 20 minutes average PET + anatomical CT image with overlayed volumes of interest. (B-D) First image shows the position of the implant on the 20 minutes average image, followed by 1 minute dynamic time frames from the same coronal slide, showing the temporal evolution of the tracer activity before (B), during (C), and after (D) the stimulation.

**Fig. 7.**
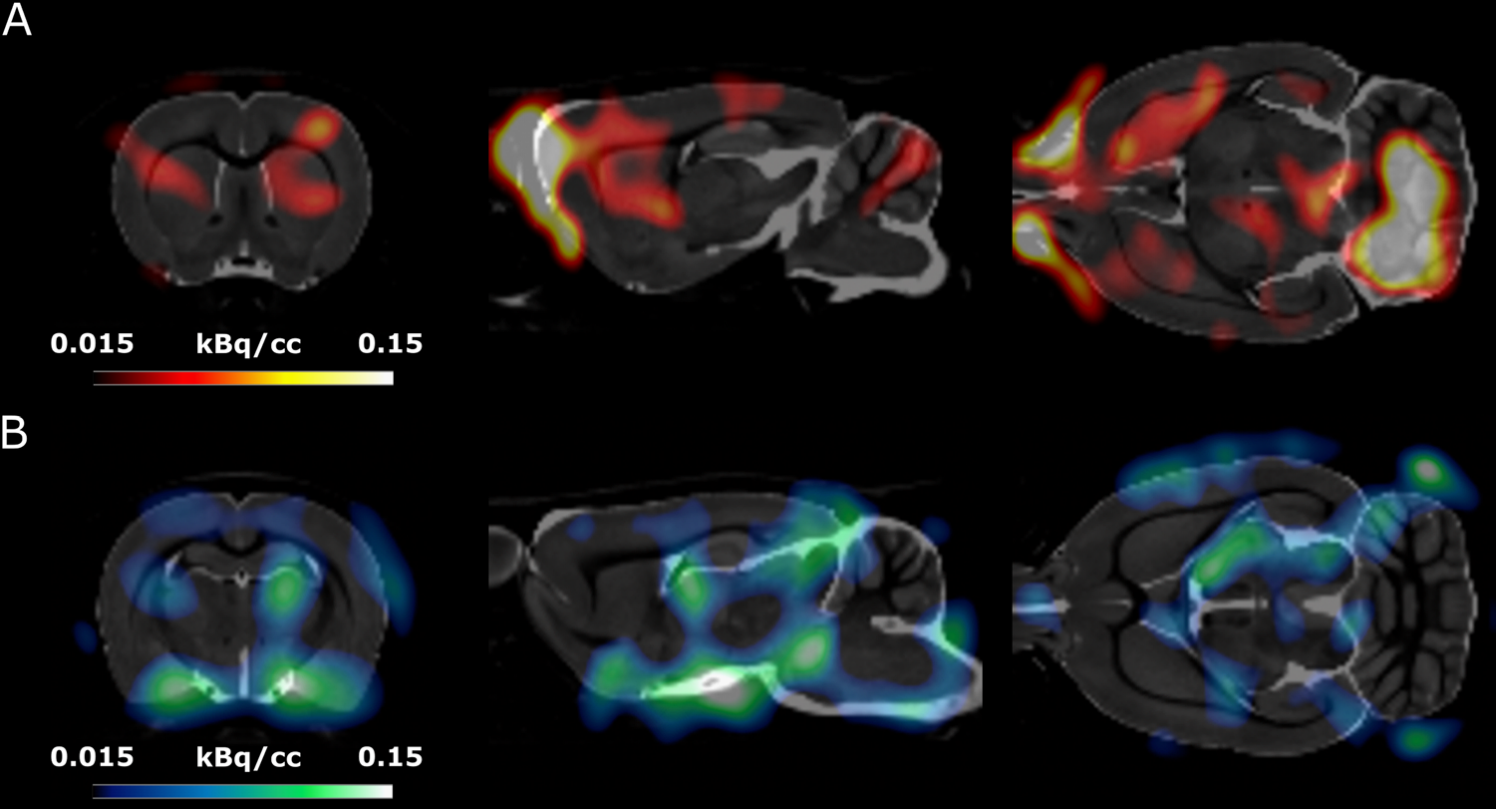
Maps of voxel difference in metabolic rate overlayed on a standard high-resolution MR for reference. (A) is focused on the dorsal striatum showing bi-lateral increase, with a seemingly higher effect ipsi-lateral to the stimulation. (B) is focused on the thalamic region showing ipsi-lateral decrease within the ventral thalamus.

These scans were used to create maps of the effect of the stimulation in single voxels thoughout the brain (Fig. 7). For easier visualization, the map has been subdivided by the type of effect (increased metabolic activity inFig. 7Aand decreased metabolic activity inFig. 7B) and overlayed to a standard high-resolution structural MR. From these maps, it is possible to observe a clear increased activity in the ipsi- and contralateral dorsal striatum (Fig. 7A), with the ipsi-lateral dorsal striatum showing seemingly higher increase than the contra-lateral. Conversely, there is a clear decreased activity in the ipsi-lateral thalamus (Fig. 7B) with no changes in the contra-lateral thalamus.

### 3.3 Metabolic effect of INS on the striatum

**Fig. 8.**
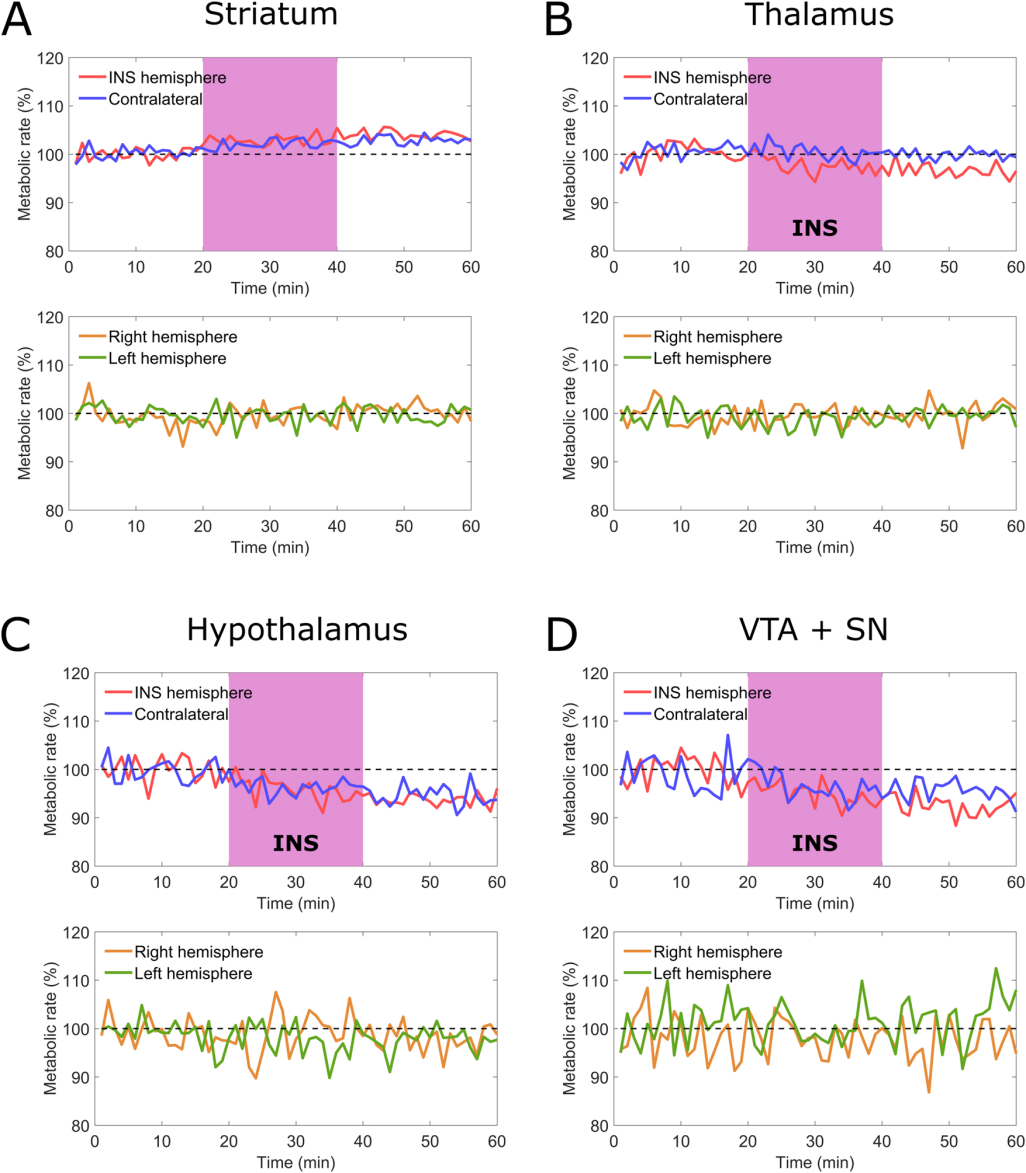
Evolution of the metabolic rate in time in different deep brain regions for the rats in the INS group (n = 3, blue and red lines) before, during, and after stimulation. The metabolic rate is compared with the one of the rats in the baseline group (n = 3, orange and green lines).*The considered regions are striatum (A), thalamus (B), hypothalamus (C), and cumulative VTA and SN (D)*.

As shown inFigure 8A, the glucose metabolic rate measured in the striatum that was directly targeted by stimulation (right hemisphere) resulted to be visibly higher than the whole-brain average for the rats in the INS group. For rats in the baseline group, instead, the metabolic rate of the right striatum was aligned to the whole-brain average. Furthermore, the acceleration of the glucose metabolism is coincident in time to the onset of the stimulation, showing a temporal correlation between the INS and the measured effect which is consistent with the preliminary electrophysiological data presented inFigure 3. After the end of the stimulation, the metabolic rate remains stable but elevated, showing a protracted effect of INS. Moreover, we observed a similar metabolic rate variation in the contralateral striatum which is present in the INS group animals and not in the baseline group animals (Fig. 8A).

### 3.4 Deep brain involvement

As presented inFigure 8B, in the animals belonging to the INS group, we observed a decrease of the thalamic activity in the hemisphere ipsilateral to the INS location, indicating that the infrared stimulus could be favoring the indirect pathway with respect to the direct one. Outside the brain regions typically involved with the CSTC circuit, we also observed a reduction of the bilateral activity in both the hypothalamus and in the region corresponding to the cumulative activity of the ventral tegmental area (VTA) and substantia nigra (SN) (Fig. 8C, D). As for the striatal effect described in the previous section, no change of metabolic rate during the scan was observed in the animals in the baseline group. In all the considered regions, the temporal coincidence between the change in metabolic rate and the onset of the stimulus confirms that the observed alterations in brain activity can be attributed to INS.

### 3.5 INS-induced recruitment of cortical brain regions

**Fig. 9.**
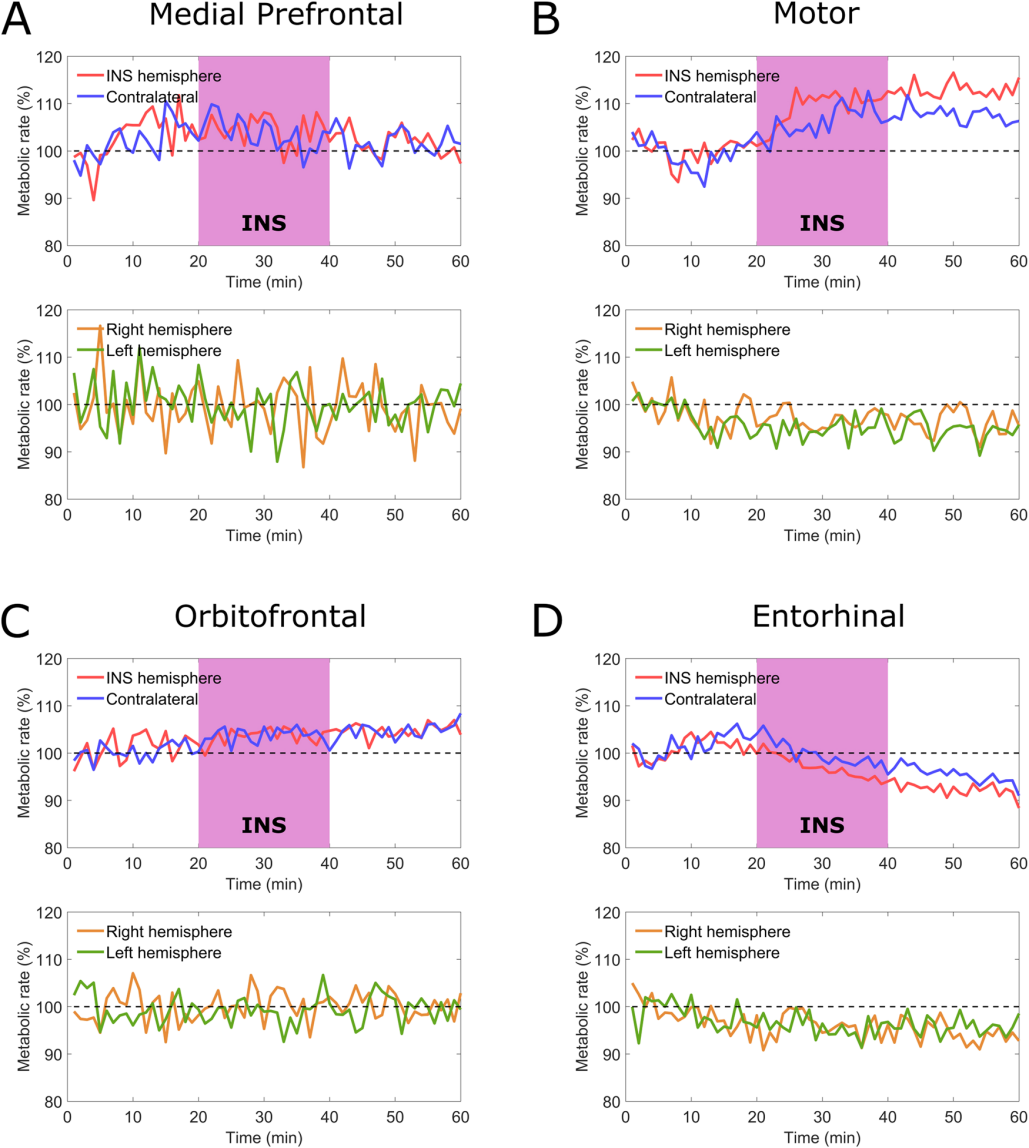
Evolution of the metabolic rate in time in different cortical regions for the rats in the INS group (n = 3, blue and red lines) before, during, and after stimulation. The metabolic rate is compared with the one of the rats in the baseline group (n = 3, orange and green lines).*The considered regions are medial prefrontal cortex (A), motor cortex (B), orbitofrontal cortex (C), and entorhinal cortex (D)*.

While the stimulation of the indirect pathway in the CSTC circuit and the related inhibition of thalamic activity are expected to correspond to an equivalent inhibition of activity in the PFC, the FDG signal measured in the medial PFC was affected by a level of noise too high to be able to discriminate variations in the metabolism of this region between the INS group and the baseline group (Fig. 9A). Despite the measured reduction in the metabolism of the thalamus, we observed a bilateral increase in the activity in the motor cortex for the animals undergoing stimulation, which was particularly pronounced in the hemisphere ipsilateral to the stimulation (Fig. 9B). Interestingly, the INS stimulus also affected the activity of both orbitofrontal and entorhinal cortex (Fig. 9C, D).

## 4 Discussion

### 4.1 Dorso-striatal effects of the stimulation

The DS has been chosen as the stimulation target for this pilot investigation due to its involvement in the CSTC circuit. Neural activity within this region was successfully initiated by our INS protocol, as confirmed by the increase in its metabolic rate ipsilateral to the stimulus (Fig. 8A). Due to the yet unclear biophysical mechanisms of INS, one must assume the stimulation to give rise to a combination of effects from the different subtypes of neurons within the DS (interneurons, glutamate projection neurons, dopamine projection neurons, andγ-aminobutyric acid (GABA)-ergic spiny projection neurons) (Kreitzer, 2009). However, since FDG-PET measures the cumulative activity of all neurons in the region of interest, it is safe to mainly attribute the recorded variations in the signal to an increased activity of GABAergic spiny projection neurons, which compose 95%of the neural population within the DS (Graveland & DiFiglia, 1985;Kreitzer, 2009). The activation of these neurons could be due to either direct stimulatory effect of INS or increased release of glutamate. The simultaneous metabolic rate increase observed in the striatum contalateral to the stimulus is consistent with existing literature that depicts a pronounced bilaterality of functional connectivity involving the striatum within circuits in the basal ganglia system (Van Den Berge et al., 2017).

### 4.2 Effects of the stimulation within the CSTC circuit

Within the CSTC circuit, the role of the DS involves two kinds of GABAergic spiny projection neurons: D1 receptor positive and D2 receptor positive (Kreitzer, 2009). These correspond to the direct and indirect pathways of the circuit, respectively. These two pathways have opposing effects on the stimulatory outcome in the thalamus, where activation of the direct pathway induces a disinhibition and thus stimulatory effect and the indirect pathway induces inhibition (Karas et al., 2019). Here, we observe a reduced thalamic activity, which suggests a stimulation of the indirect (D2 positive) pathway, which is different from what we observed in our previous study, where no changes in either region were observed following selective dopamine release (Casado-Sainz et al., 2022). However, it is important to consider that the thalamus is a highly complex structure divided into many subnuclei that are responsible for projections to distinct regions in the whole cortex (Habas et al., 2019). Here, we can only report the overall change in activity, mainly consisting of glutamatergic neurons projecting to cortical and striatal regions, with the given limit of resolution that PET provides (Roy et al., 2022). Despite the decrease in thalamic activity, we observed a bilateral increase in the metabolism of the motor cortex. This seemingly counterintuitive effect could be correlated to the use of isoflurane, a GABA-A receptor positive allosteric modulator, as anesthesia (Shelton & Nicholson, 2010), since GABA is one of the major neurotransmitters in the thalamus (Quiñones et al., 2021). However, the result is also in line with previous research showing an increased cortical activity following pharmacological activation of the indirect pathway (Servaes et al., 2016) and reduction in motor activity related to thalamic deep brain stimulation (Casagrande et al., 2019).

### 4.3 Distal effects outside the CSTC circuit

While the main focus of this study was the interrogation of the CSTC circuit using a combination of INS and FDG-PET, we observed an effect of the stimulation in brain regions that do not fit within the classical description of that circuit. This hints to the involvement of other striato-cortical circuits as a result of the delivered INS stimulus. The INS-induced variation in the bilateral activity of the hypothalamus and cumulative VTA and SN, visible inFigure 8C and D, in particular, seems to indicate the involvement of cortico-striato-hypothalamic limbic circuits involved in feeding behavior, despite their usual association with the ventral striatum (VS) rather than the DS (Averbeck & Murray, 2020;Clarke et al., 2018). This is further reinforced by the observed bilateral variation in the activity of both the orbitofrontal and entorhinal cortexes (Fig. 9C, D), which are a part of these limbic circuits (Averbeck & Murray, 2020). It is, however, not possible within this study to discriminate if this is caused by the interaction between the nigrostriatal and mesocortical dopamine pathways (Wise, 2009).

### 4.4 Effect size of the observed metabolic variations

**Fig. 10.**
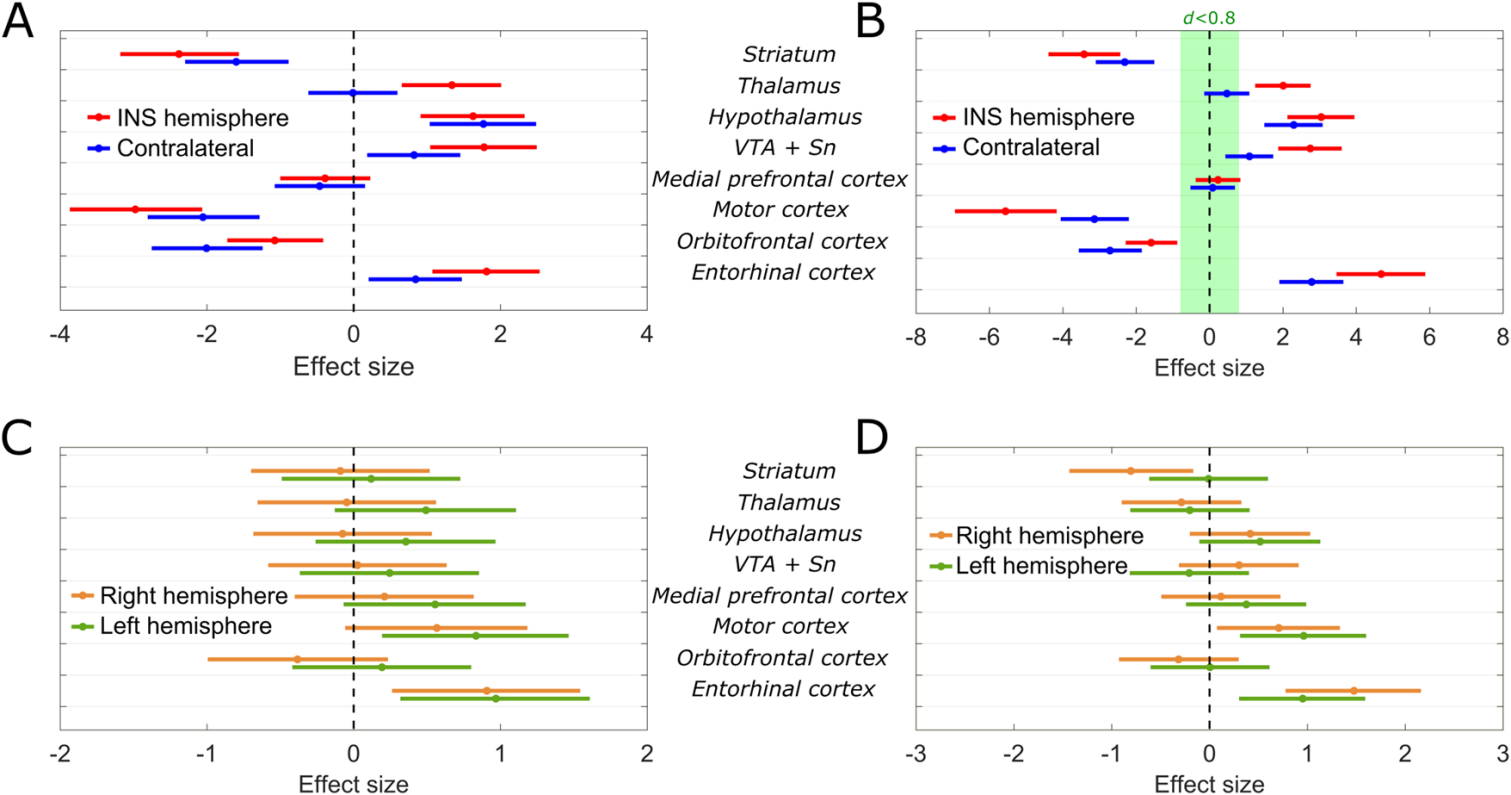
(A, B) Effect size, estimated by Cohen’s*d*, of the metabolic activity variation with respect to the baseline period for the stimulation and poststimulation periods, respectively (INS group, N = 3). The green shaded area in (B) represents the region outside which effects can be considered as large. (C, D) Effect size, estimated by Cohen’s*d*, of the metabolic activity variation with respect to the first 20 minutes period for the second and third and 20 minutes periods, respectively (baseline group, N = 3).

To further validate the results of this proof-of-concept investigation, the effect size of the changes in brain activity discussed in the previous section has been investigated for the animals in the INS group. In order to do so, the mean value and standard deviation of the normalized metabolic rates recorded in individual brain regions, averaged over the three animals in the group, has been calculated for the baseline (first 20 minutes), stimulation (second 20 minutes), and poststimulation (last 20 minutes) time periods of the dynamic scans. These have then been used to calculate, via Cohen’s*d*, the effect size of the metabolic variations observed during the stimulation and poststimulation periods with respect to the baseline (Table 1). Here, due to the relatively slow rate of the increase or decrease of the activity during the stimulation period, the effect size calculated by comparing the poststimulation and baseline periods can be regarded as a better indicator of the significance of the observations. However, for both comparisons, we can report Cohen’s*d*values larger than 0.8 (typically defined as the threshold above which an effect can be considered large (Chen et al., 2010)) for all the metabolic effects of INS described in the previous sections. The validity of our observations is further underlined by an evaluation of the 95% confidence intervals of the calculated effect sizes, presented for the INS group inFigure 10A and B, with positive values ofdcorresponding to a reduction in the activity and negative values corresponding to an increase. For all the effects described in the previous sections as significant variations, the absolute value of the Cohen’s*d*is>0with 95% confidence. Furthermore, when comparing the poststimulation and baseline periods, for almost all of these variations we can define the effect as large (d>0.8) with the same high degree of confidence (Fig. 10B). In contrast, for animals belonging to the baseline group, almost no significant metabolic variations can be observed when comparing the second and third 20 minutes periods of the scan to the first one (Fig. 10C, D). The few exceptions, which can be attributed to the small size of the group or to a reduction of activity in specific brain regions (e.g. motor cortex) due to the effect of the anesthesia, anyway exhibit values ofdseveral times lower when compared to the INS group. In the case of the motor cortex, in particular, the opposite sign ofdbetween the baseline and INS groups further strengthens the observation of an INS-induced increase in activity.

**Table 1.** Mean value, standard deviation, and effect size comparing the baseline (0-20 minutes), stimulation (20-40 minutes), and poststimulation (40-60 minutes) normalized activity in different brain regions of the rats in the INS group.

Brain region	Hemisphere	Baseline		Stimulation		Poststimulation		Baseline-Stimulation effect size	Baseline-Poststimulation effect size
		Mean	Stdev	Mean	Stdev	Mean	Stdev		
Striatum	Left	100.0	1.2	101.8	0.9	102.5	0.9	1.6	2.3
	Right	100.0	1.3	103.0	1.1	103.9	0.9	2.4	3.4
Thalamus	Left	100.0	1.6	100.0	1.5	99.3	1.0	0.01	0.5
	Right	100.0	2.0	97.5	1.6	96.4	1.4	1.3	2.0
Hypothalamus	Left	100.0	2.3	96.6	1.5	94.9	2.1	1.8	2.3
	Right	100.0	2.5	96.0	2.4	93.8	1.3	1.6	3.0
VTA+Sn	Left	100.0	3.7	97.3	2.6	96.7	2.0	0.8	1.1
	Right	100.0	2.9	95.3	2.3	92.9	2.1	1.8	2.7
Medial prefrontal cortex	Left	100.0	3.7	101.7	3.3	99.8	2.7	0.5	0.08
	Right	100.0	4.8	101.6	3.1	99.1	2.5	0.4	0.2
Motor cortex	Left	100.0	3.2	106.7	3.2	108.1	1.6	2.1	3.1
	Right	100.0	2.7	109.3	3.4	112.7	1.7	3.0	5.6
Orbitofrontal cortex	Left	100.0	1.7	103.4	1.6	104.5	1.5	2.0	2.7
	Right	100.0	2.8	102.5	1.6	103.6	1.4	1.1	1.6
Entorhinal cortex	Left	100.0	2.4	97.9	4.4	94.0	1.7	0.8	2.8
	Right	100.0	2.1	95.9	2.3	91.4	1.4	1.8	4.7

## 5 Conclusions

We demonstrated the feasibility of using a combination of INS and FDG-PET for mapping neural functional networks*in vivo*on a whole-brain scale. By using broadband supercontinuum IR light combined with custom-made soft bi-directional neural interfaces, we stimulated the dorsal striatum in anesthetized animals to evaluate the functionality of this novel approach on the CSTC circuit. We obtained a temporal profile of the glucose metabolism in different brain regions by scanning at regular (1 minute) intervals. This allowed us to temporally correlate the variation of the metabolic rate in all considered regions with the onset of the INS stimulus, thus excluding the possibility of the effects being caused by the implantation, since any change in activity caused by the surgical procedure could be reasonably expected to appear in the pre-stimulus recording period.

The stimulation protocol with 2 minutes illumination periods, which was already validated by electrophysiological recordings in our previous study (Meneghetti et al., 2023), was confirmed by the increase of the metabolic rate in the stimulated region. Whole-brain scale mapping of the glucose metabolism during the stimulation allowed to observe a rich network of connections in both the ipsi- and contralateral hemispheres. This involvement of regions distal to the stimulated areas spanned from the cortex to the deep brain. Interestingly, this included regions not typically considered as a part of the CSTC circuit, which was the focus of this study. The presence of bilateral variation in the hypothalamic region, as well as in the orbitofrontal and entorhinal cortex suggest the additional involvement of the cortico-striato-hypothalamic circuitry. An effect size estimation through Cohen’s*d*revealed all of the described variations to be significantly strong, especially when comparing the poststimulation periods to the baseline ones.

In summary, INS and PET can be combined to form a powerful mapping tool for brain circuitry*in vivo*. The high spatial specificity and lack of need for viral manipulation of INS, combined with the versatility of molecular targets for PET, can provide a powerful tool to rapidly study brain connectivity with high precision while decoding the studied networks on a chemical level. Novel infrared interfaces based on soft materials, as the ones used in this study, open possibilities for chronic implantations with minimal inflammation and, subsequently, for studies in behaving animals. We believe this work will pave the way to future investigations in different subcortical brain region and thus represent a cornerstone for further advances in our understanding of the inner mechanisms of the brain.

## Data Availability

All software and procedures concerning the data acquisition and analysis have been detailed in theMaterials and Methods section. All data sets are available from the corresponding author upon reasonable request.
